# Identification of *EP300* as a Key Gene Involved in Antipsychotic-Induced Metabolic Dysregulation Based on Integrative Bioinformatics Analysis of Multi-Tissue Gene Expression Data

**DOI:** 10.3389/fphar.2021.729474

**Published:** 2021-08-13

**Authors:** Albert Martínez-Pinteño, Patricia Gassó, Llucia Prohens, Alex G. Segura, Mara Parellada, Jerónimo Saiz-Ruiz, Manuel J. Cuesta, Miguel Bernardo, Amalia Lafuente, Sergi Mas, Natalia Rodríguez

**Affiliations:** ^1^ Department of Basic Clinical Practice, Pharmacology Unit, University of Barcelona, Barcelona, Spain; ^2^ Institut d’Investigacions Biomèdiques August Pi i Sunyer (IDIBAPS), Barcelona, Spain; ^3^ Centro de Investigación Biomédica en Red de Salud Mental (CIBERSAM), Instituto de Salud Carlos III, Madrid, Spain; ^4^ Department of Child and Adolescent Psychiatry, Hospital General Universitario Gregorio Marañón, School of Medicine, Universidad Complutense, IiSGM, Madrid, Spain; ^5^ Department of Psychiatry, Hospital Universitario Ramón y Cajal, IRYCIS, Universidad de Alcalá, Madrid, Spain; ^6^ Department of Psychiatry, Complejo Hospitalario de Navarra, Instituto de Investigación Sanitaria de Navarra (IdiSNA), Pamplona, Spain; ^7^ Barcelona Clínic Schizophrenia Unit, Hospital Clínic de Barcelona, Barcelona, Spain; ^8^ Department of Medicine, University of Barcelona, Barcelona, Spain

**Keywords:** antipsychotics, weight gain, metabolic syndrome, pharmacogenetics, EP300, gene, microarray, gene expression

## Abstract

Antipsychotics (APs) are associated with weight gain and other metabolic abnormalities such as hyperglycemia, dyslipidemia and metabolic syndrome. This translational study aimed to uncover the underlying molecular mechanisms and identify the key genes involved in AP-induced metabolic effects. An integrative gene expression analysis was performed in four different mouse tissues (striatum, liver, pancreas and adipose) after risperidone or olanzapine treatment. The analytical approach combined the identification of the gene co-expression modules related to AP treatment, gene set enrichment analysis and protein-protein interaction network construction. We found several co-expression modules of genes involved in glucose and lipid homeostasis, hormone regulation and other processes related to metabolic impairment. Among these genes, *EP300*, which encodes an acetyltransferase involved in transcriptional regulation, was identified as the most important hub gene overlapping the networks of both APs. Then, we explored the genetically predicted *EP300* expression levels in a cohort of 226 patients with first-episode psychosis who were being treated with APs to further assess the association of this gene with metabolic alterations. The *EP300* expression levels were significantly associated with increases in body weight, body mass index, total cholesterol levels, low-density lipoprotein cholesterol levels and triglyceride concentrations after 6 months of AP treatment. Taken together, our analysis identified *EP300* as a key gene in AP-induced metabolic abnormalities, indicating that the dysregulation of *EP300* function could be important in the development of these side effects. However, more studies are needed to disentangle the role of this gene in the mechanism of action of APs.

## Introduction

Antipsychotics (APs) are the primary medication used for the treatment of schizophrenia and other psychiatric disorders. These medications, especially second-generation APs, are associated with a wide range of side effects including weight gain and other metabolic alterations that can lead to the development of metabolic syndrome, type 2 diabetes (T2D) and serious cardiovascular diseases (Fernandez-Egea et al., 2011; Foley and Morley, 2011; De Hert et al., 2012; Ward et al., 2013; Tek et al., 2016). The risk of metabolic adverse events varies among the APs, with olanzapine and clozapine having the highest risk of causing such effects and others having an intermediate (e.g., risperidone) or low risk (e.g., aripiprazole) (Lett et al., 2012; Pillinger et al., 2020). Even in the absence of AP treatment, individuals with psychiatric diseases are more likely to suffer obesity and metabolic disorders than the general population, with drug therapy contributing to the increase in these rates and aggravating the metabolic profile in these patients (Holt and Peveler, 2009; Mitchell et al., 2013; Correll et al., 2014). The associated co-morbidities reduce quality of life and increase patient mortality (Kritharides et al., 2017). Furthermore, these side effects are a major cause of treatment discontinuation, thus, worsening prognosis and increasing the risk of relapse (Doane et al., 2020). Therefore, the identification of robust predictors of AP-induced side effects could contribute to the identification of those subjects at a higher risk of developing metabolic disturbances, facilitating better treatment selection and improving clinical outcomes.

Although the underlying factors involved in AP-induced metabolic dysregulation are not well understood, genetic factors are assumed to be important based on the findings from twin and sibling studies (Gebhardt et al., 2010). Thus, several pharmacogenetic studies have been conducted, most of them using a candidate gene approach, which is limited by our current understanding of the mechanism of action of APs (Mas et al., 2012, 2015). Evidence suggests that the antagonistic effects of APs on neurotransmitter receptors involved in the control of food intake, such as the histamine H1 receptors (H1R), the serotonin 2C receptors (5-HT_2C_) and the dopamine D2 receptors (D2DR), are closely linked to cardiovascular risk and metabolic dysfunction (De Hert et al., 2012; Reynolds and McGowan, 2017). However, the exact downstream mechanisms that may promote the development of metabolic abnormalities in patients receiving APs remain elusive. To date, by using this candidate gene approach, several susceptibility genes of weight gain and other metabolic adverse effects induced by APs have been identified. These genes are mostly related to neurotransmitter systems, food intake regulation and energy metabolism, and include *ADR2A*, *BDNF*, *CNR1*, *DRD2*, *DRD3*, *FTO*, *GNB3*, *HTR2C*, *INSIG2*, *LEP*, *LEPR*, *MC4R*, *MTHFR*, *NPY*, *SNAP25*, and *SREBF1*, among others (Arranz et al., 2011; Shams and Müller, 2014; Devlin and Panagiotopoulos, 2015; MacNeil and Müller, 2016; Zhang et al., 2016; Zhang and Malhotra, 2018; Gassó et al., 2020; Yoshida and Müller, 2020). A meta-analysis of 72 articles with a combined sample of 6700 patients from 46 non-overlapping cohorts found that *ADRA2A*, *DRD2*, *HTR2C*, and *MC4R* polymorphisms were some of the variants significantly associated with AP-induced weight gain, and were those with the largest effect sizes (Zhang et al., 2016). Genome-wide association studies (GWAS) have also addressed this issue, suggesting novel variants associated with AP-induced metabolic effects, such as those located in *CIDEA*, *DGKB*, *MC4R*, *MEIS2*, *PRKAR2B* and *PTPRD*, among others (Adkins et al., 2011; Malhotra et al., 2012; Brandl et al., 2016; Yu et al., 2016; Maciukiewicz et al., 2019; Corfitsen et al., 2020; ter Hark et al., 2020). Despite this associations, only few genes have been confirmed in independent studies. Hence, further research is required to identify robust markers and validate their values as predictors of AP-induced metabolic disturbances before their implementation in daily clinical practice (Arranz et al., 2021; Islam et al., 2021).

Here, we aimed to investigate the molecular pathways underlying AP-induced metabolic effects and identify potential candidate genes for future pharmacogenetic studies using a translational gene expression study from mice to humans. First, whole-genome expression changes were analyzed in different mouse tissues after treatment with olanzapine or risperidone, two APs with high and intermediate metabolic risks associated, respectively (Lett et al., 2012; Pillinger et al., 2020), that are considered the most commonly prescribed APs worldwide (Hálfdánarson et al., 2017). We focused on analyzing the gene expression and their related biological processes that are modified by both APs in order to investigate the overlapping genes and mechanisms of action involved in AP-induced metabolic imbalance. Then, we explored the genetically predicted gene expression levels of the key genes identified in a cohort of patients with first-episode psychosis (FEP) who were being treated with APs to further assess the association of these genes with metabolic alterations.

## Materials and Methods

A detailed description of the methods used in the present study is available in Supplementary Material.

### Animals and Drug Treatment

Mice were chronically treated for 28 days with a daily subcutaneous dose of vehicle (saline containing 5% dimethylsulfoxide (DMSO) and 5% Tween 20), risperidone (1 mg/kg) or olanzapine (3.5 mg/kg). Each group included 8 C57BL/6JOlaHsd mice (4 males and 4 females). The animals were maintained with *ad libitum* access to food and water, using a standard chow diet.

All animal-related procedures were performed in accordance with the European Union guidelines for the care and use of laboratory animals and were approved by the Animal Care Committee of the University of Barcelona and by the Generalitat de Catalunya.

### Metabolic Assessment in Mice

Several metabolic parameters (including body weight, blood glucose levels, total cholesterol levels and triglyceride levels) were assessed in all mice at baseline (1 week before the start of AP treatment) and after 28 days of treatment. Fasting glucose levels were measured using a glucometer AlphaTRAK^®^ 2 (Zoetis, Parsippany, NJ, United States). Total cholesterol levels and triglyceride levels were measured using the Cholesterol Liquid assay kit and the Triglycerides Liquid assay kit, respectively (Química Clínica Aplicada S.A., Amposta, Spain). The percentage of change for each parameter between both measures was calculated. A detailed description of the methods used for the evaluation of metabolic parameters is available in Supplementary Material.

### RNA Isolation and Microarray Hybridization

After 28 days of treatment, animals were sacrificed by decapitation 1 h after the last injection. Then, their tissues were rapidly removed and placed on ice. Four tissues that are potentially relevant for the development of AP-induced metabolic effects were used: the liver, the pancreas, visceral adipose tissue and the striatum. Total RNA was isolated using Trizol reagent (Life Technologies, Foster City, CA, United States) and was further purified using the miTotal RNA Extraction Miniprep System (Viogene Biotek Corp, New Taipei City, Taiwan). The RNA samples were then submitted to the Kompetenzzentrum für Fluoreszente Bioanalytik Microarray Technology (KFB, BioPark Regensburg GmbH, Regensburg, Germany) for labeling and hybridization to the Affymetrix mouse Clariom S Arrays (Affymetrix, Inc., Santa Clara, CA, United States).

### Microarray Data Analysis

A weighted gene co-expression network analysis (WGCNA) was used to identify modules of highly co-expressed genes significantly associated (*p*-value < 0.05) with AP treatment (risperidone or olanzapine) (Langfelder and Horvath, 2008). Then, a gene set enrichment analysis of these co-expression modules was performed using FatiGO (Al-Shahrour et al., 2007) and Gene Ontology (GO) terms (Ashburner et al., 2000). From the significantly enriched (false discovery rate (FDR)-corrected *p*-value < 0.05) biological processes, the GO terms related to metabolic processes and metabolic dysregulation were selected for further analysis. Genes included in the selected GO terms were used to create a single protein-protein interaction (PPI) network for each AP using the SNOW program (Minguez et al., 2009). A single PPI network integrating both APs was also constructed by merging the networks obtained independently for risperidone and olanzapine using the intersection option in Cytoscape 3.7.2 (Shannon et al., 2003). For each node, the degree of connectivity was calculated to identify the candidate hub genes from the constructed PPI networks.

Full details of the extraction, labeling and hybridization protocols as well as the raw array data (.cel files) and the pre-processed data matrix are available at the Gene Expression Omnibus database (http://www.ncbi.nlm.nih.gov/geo/; accession number GSE180473).

### Exploration of the Hub Genes in a Naturalistic Cohort of Patients With FEP

#### Participants

The sample comprised 226 patients with FEP (age 23.6 ± 6.0 years; 66.8% were males) recruited in the PEPs study. The participants were prescribed at least one second-generation AP during the 6-month follow-up period. During the follow-up period, about 40% of the patients were treated with olanzapine or clozapine, which have the highest risk of inducing weight gain or worsening metabolic parameters, while more than 50% were administered APs with an intermediate or low risk of causing such side effects, with risperidone and aripiprazole being the most frequently prescribed in this group. Further details of the study population are described in the Supplementary Material. The detailed protocol of the PEPs study has been published elsewhere (Bernardo et al., 2013; Bioque et al., 2016). The study was approved by the ethics committees of all the participating clinical centers. Informed consent was obtained from all the participants. In the case of children under 16 years of age, parents or legal guardians gave written informed consent before study participation, while the patients themselves also agreed to participate.

#### Metabolic Assessment

Several anthropometric and metabolic traits were assessed at baseline and at 6 months, including body weight, body mass index (BMI), blood glucose levels, total cholesterol levels, low-density lipoprotein (LDL) cholesterol levels, high-density lipoprotein (HDL) cholesterol levels and triglyceride (TG) levels. The percentage of change for each parameter between both visits was calculated (Table 1).

**TABLE 1:** Metabolic data of the study participants at baseline and at the 6-months follow-up.

	N	Baseline	6 months	% Gain
Weight (kg), mean ± SD	226	69.18 ± 14.58	73.77 ± 14.62	8.65 ± 11.17
BMI, mean ± SD	225	23.49 ± 4.70	25.13 ± 4.19	8.60 ± 11.20
Glucose (mg/dl), mean ± SD	206	83.98 ± 15.80	85.52 ± 14.35	3.39 ± 16.28
Cholesterol (mg/dl), mean ± SD	212	160.85 ± 36.61	167.09 ± 36.36	6.36 ± 25.44
LDL cholesterol (mg/dl), mean ± SD	171	94.53 ± 29.09	98.97 ± 31.98	3.98 ± 30.340
HDL cholesterol (mg/dl), mean ± SD	172	49.19 ± 12.46	47.94 ± 13.02	1.38 ± 24.46
Triglycerides (mg/dl), mean ± SD	205	87.84 ± 49.18	102.10 ± 58.81	27.53 ± 66.98

BMI, body mass index; HDL, high-density lipoprotein; LDL, low-density lipoprotein.

#### Genotyping and Gene Expression Prediction

Samples from all the individuals in the study were genotyped at the Centro Nacional de Genotipado (CeGen, Santiago de Compostela, Spain), using the Affymetrix Axiom Spain Biobank Array. The genotyping data were called using the Axiom Analysis Suite (Mas et al., 2020) and were then submitted to the Michigan Imputation Server (Das et al., 2016), following the standard pipeline for the Minimac4 software. Genotyping data were used to predict the genetically regulated gene expression levels for each individual through the gene expression imputation method, PrediXcan (Gamazon et al., 2015). Gene expression of the previously identified hub genes was predicted in five different tissues that are potentially relevant for the development of AP-induced metabolic alterations, including the small intestine, the pancreas, the liver, visceral adipose tissue and subcutaneous adipose tissue, following the standard procedure found at https://github.com/hakyimlab/PrediXcan.

### Statistics

Data were analyzed using IBM SPSS Statistics version 20.0 (IBM Corp, Chicago, IL, United States). Normality was assessed using Kolmogorov-Smirnov and Shapiro-Wilk tests. Means ± standard deviations (SD) were computed for continuous variables. The differences between groups in the metabolic parameters assessed in mice were analyzed using analysis of variance. As some differences between males and females were observed in some of the parameters evaluated, the analysis was adjusted for gender.

For the analysis of the hub genes identified in the cohort of patients with FEP, the predicted gene expression levels of the *EP300* gene were categorized as low, medium or high according to the three values of gene expression obtained in each tissue (−0.13, −0.07, and 0.00, respectively). Differences in metabolic variables between these categories were tested using analysis of variance. The analysis was adjusted for sociodemographic and clinical variables that might affect the parameters assessed, including gender, age, and the type of AP according to its potency (high, low, or no risk of increasing weight or worsening metabolic parameters). A further pairwise comparison analysis was performed using Bonferroni’s post-hoc test.

## Results

**FIGURE 1:**
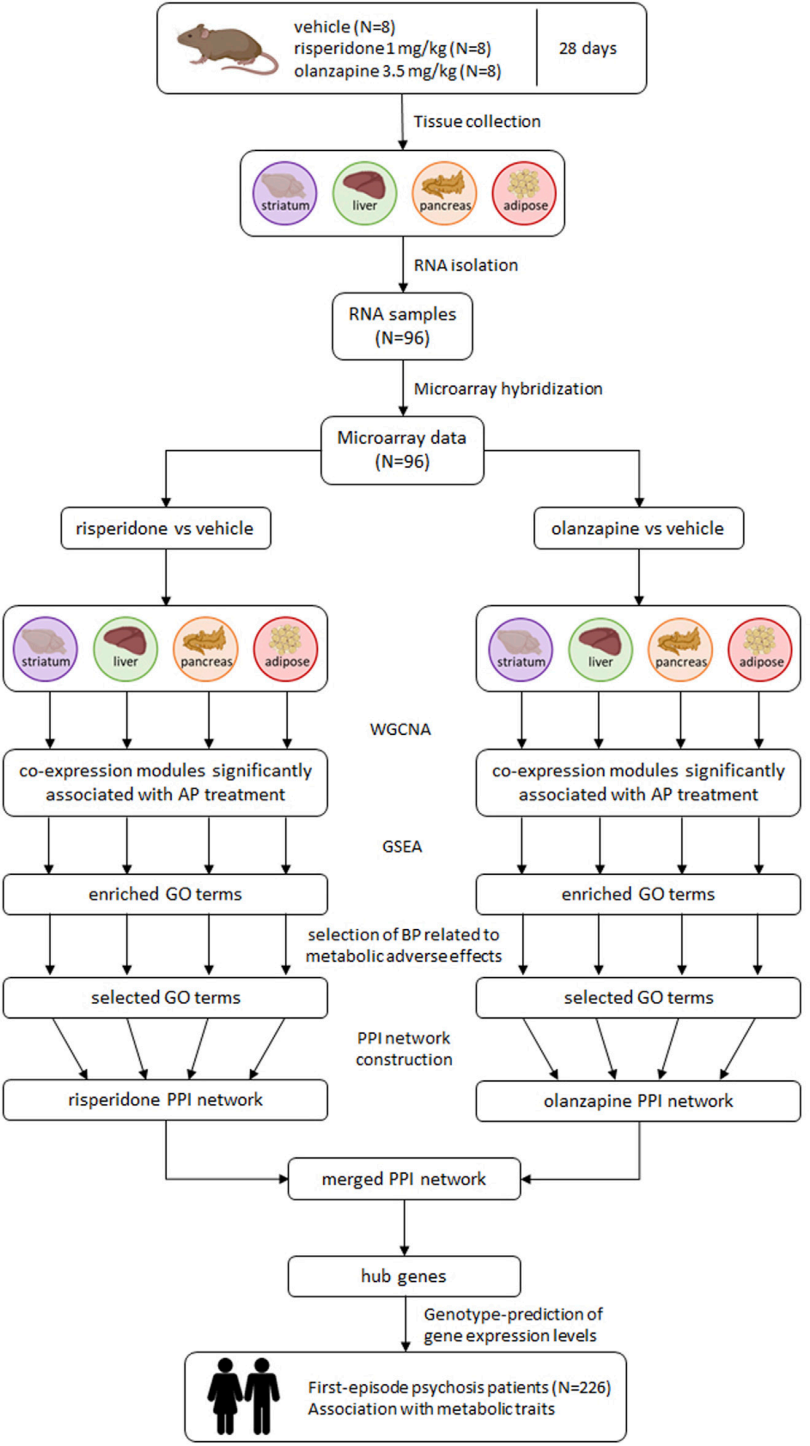
Overview of the analysis procedure followed in the study. AP: antipsychotic; BP: biological processes; GO: Gene Ontology; GSEA: gene set enrichment analysis; PPI: protein-protein interaction; WGCNA: weighted gene co-expression network analysis.

Figure 1 shows an overview of the analysis procedure followed in the present study.

An increase in body weight (15.56% ± 5.25), fasting glucose levels (28.10% ± 43.95) and triglyceride levels (96.52% ± 89.50) was observed in mice after 28 days of treatment while cholesterol levels remained mainly unaltered (−1.56 ± 13.15). No significant differences were identified between the different treatment groups.

Regarding the gene expression analysis, 423 modules of co-expressed genes were obtained in the risperidone-treated mice: 114 in the striatum, 230 in the liver, 73 in the pancreas and six in the adipose tissue (Supplementary Figure S1). Twenty of these modules, containing between 37 and 179 genes, were significantly associated with risperidone treatment: three of those in the striatum, 13 of those in the liver and 4 of those in the pancreas (Supplementary Table S1). Regarding the olanzapine-treated mice, 483 modules of co-expressed genes were identified in the first analysis: 65 in the striatum, 223 in the liver, 140 in the pancreas and 55 in the adipose tissue (Supplementary Figure S1). Forty-six of these modules, containing between 33 and 1841 genes, were significantly associated with olanzapine treatment: five of those in the striatum, 21 of those in the liver, 15 of those in the pancreas and five of those in the adipose tissue (Supplementary Table S1). Two of the modules (red and yellow) significantly associated with olanzapine treatment in the striatum contained a large number of co-expressed genes (1,105 and 1,841 genes, respectively); hence, from these modules, only the genes with a module membership (MM) above 0.5 and a gene significance (GS) above 0.6 were selected for further analysis. Thus, 245 and 346 genes were considered for the red and yellow modules, respectively.

**FIGURE 2:**
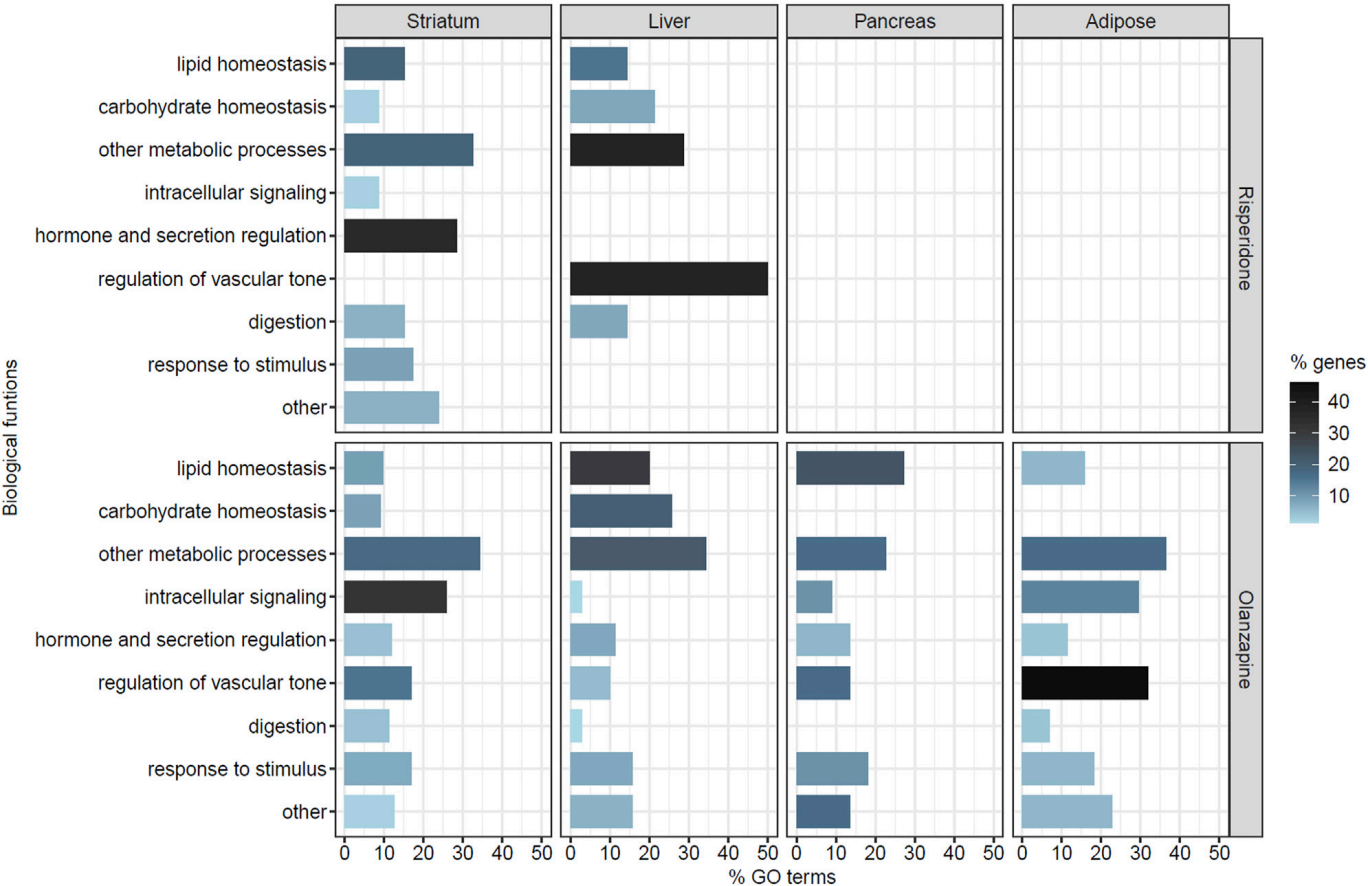
Selected biological processes related to metabolic dysregulation in each tissue of risperidone- or olanzapine-treated mice. The Y-axis represents the different categories of biological functions considered. The X-axis represents the percentage of processes (% GO terms) related to each biological function out of all the selected GO terms in each tissue for each antipsychotic. The color gradient represents the percentage of genes (% genes) related to each biological function out of all the genes included in the selected GO terms in each tissue for each antipsychotic. No significant gene modules were identified in the adipose tissue of risperidone-treated mice. None of the significant GO terms identified in the pancreas of the risperidone-treated mice were related to metabolic dysregulation.

To gain further insight into the biological functions of the genes included in each co-expression module that was significantly associated with AP treatment, a functional enrichment analysis was performed. We found significantly enriched GO terms in 14 of the 20 co-expression modules associated with risperidone treatment (2 in the striatum, 11 in the liver and one in the pancreas) and 26 of the 46 modules associated with olanzapine treatment (4 in the striatum, 13 in the liver, five in the pancreas and 4 in the adipose tissue). For both APs, the enriched biological processes were related to different functions including tissue development, cell proliferation and differentiation, intracellular signaling, immune system processes and metabolic and biosynthetic processes, among others (Supplementary Tables S2, S3). The significantly enriched GO terms related to metabolic processes and metabolic dysregulation were selected for further analysis. These biological processes were related to glucose and lipid homeostasis, substance metabolism, digestion and the regulation of nutrient levels, hormone and secretion regulation, intracellular signaling through second messengers, the regulation of vascular tone and responses to different stimuli (Figure 2). Out of all the significant GO terms identified, 61 processes (21.7%) were selected for risperidone and 341 (25.5%) for olanzapine.

The genes included in the selected GO terms (60 genes for risperidone and 263 for olanzapine) were used to create a PPI network for each AP (Supplementary Figure S2). The network constructed with the gene set for the risperidone-treated mice comprised 46 genes and included 29 (48.3%) of the 60 genes from the original list whereas the network constructed with the gene set for the olanzapine-treated mice comprised 295 genes, including 140 (53.2%) of the 263 genes from the original list. The nodes of both PPI networks showed more connections (degree of connectivity *p*-value ≤ 1 × 10^−3^), greater connectivity (clustering coefficient *p*-value < 0.01) and more hub nodes (betweenness centrality *p*-value < 0.01) than would be expected by chance. The degree of connectivity was calculated for each node and the top 10% of the genes with the highest degree were considered the hub genes of each network. Thus, seven hub genes with a degree of connectivity ranging between 5 and 10 were identified in the risperidone network, while 30 hub genes with a node degree of 11–25 were obtained in the olanzapine network.

**FIGURE 3:**
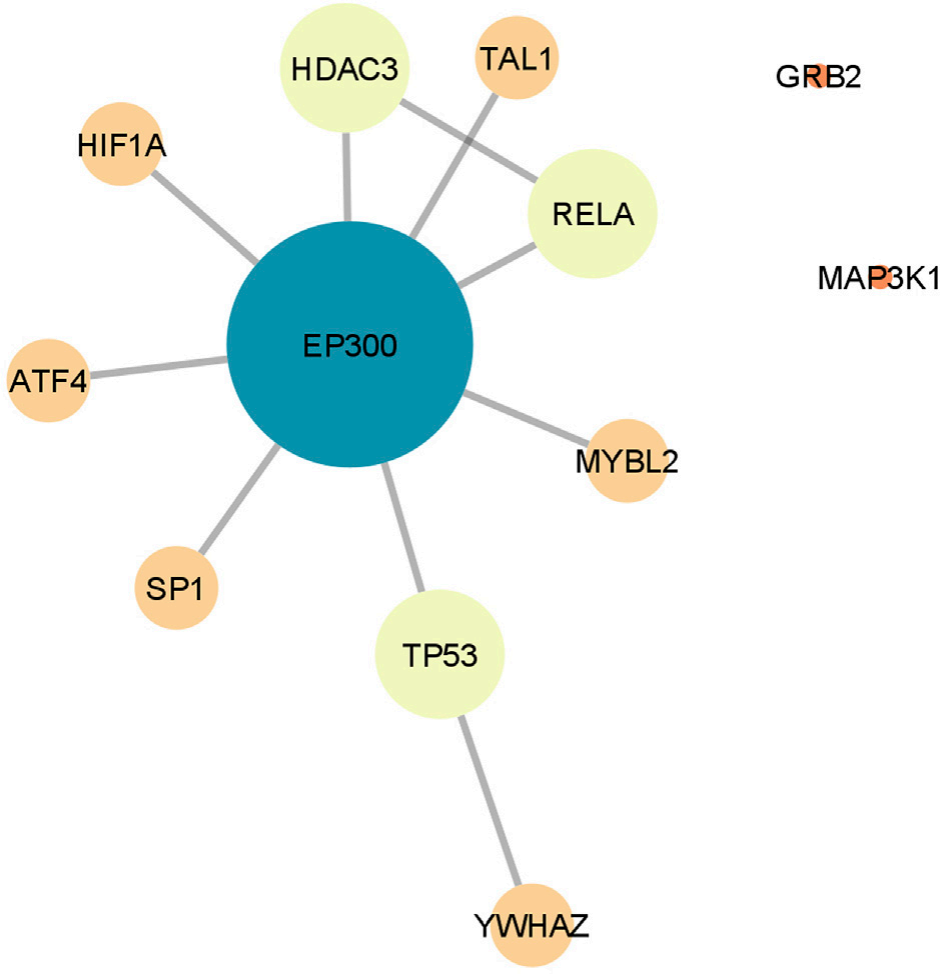
Protein-protein interaction network constructed by merging the networks derived independently for risperidone and olanzapine. Only the genes overlapping the networks of both antipsychotics are shown. Node size and color represent the number of connections (degree) of each gene.

To find the overlapping genes involved in the downstream processes regulated by both APs, a single PPI network was constructed by merging the networks obtained independently for risperidone and olanzapine (Figure 3). The resulting network showed 12 common genes, 10 of which were interconnected. The most connected gene was *EP300*, with a degree of connectivity of 8, thereby becoming the central gene of the network. *EP300* was also identified as one of the most important hub genes in the networks of both APs, showing a node degree of 10 in the risperidone network (thus, becoming the most connected gene of the network) and a degree of 21 in the olanzapine network (becoming the second most connected gene of the network).

**FIGURE 4:**
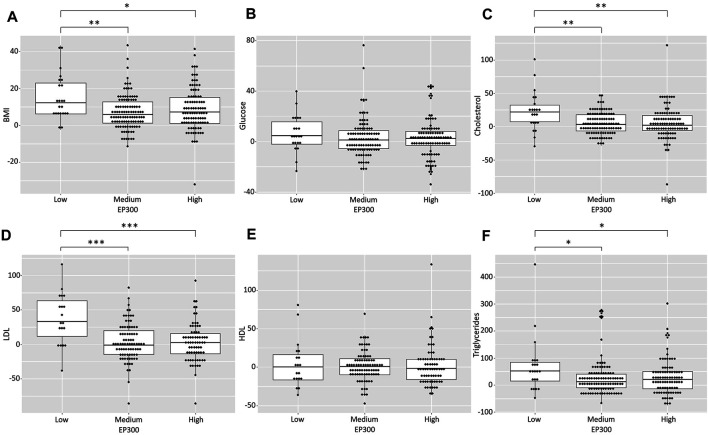
Exploration of *EP300* as a key gene in antipsychotic-induced metabolic dysregulation in patients with FEP receiving second-generation antipsychotics. Scatter box plots of the increases in the anthropometric and metabolic traits assessed according to the predicted *EP300* expression levels using the prediction model of the liver, the adipose tissue and the small intestine. *p < 0.05; **p < 0.01; ***p < 0.001.

To obtain more evidence of the role of *EP300* as a key gene in AP-induced metabolic effects, we used the genetically predicted gene expression levels in a naturalistic cohort of patients with FEP receiving APs. Two predictive models were used to impute the gene expression levels of *EP300* in different tissues based on the genotyping data: one for the prediction in the small intestine, the liver and subcutaneous adipose tissue and another one for the prediction in the pancreas and visceral adipose tissue. Then, we tested the association between the predicted gene expression values and the metabolic status of the patients at baseline and after the 6-months follow-up. No significant association was observed between the *EP300* expression levels and the anthropometric and metabolic parameters measured at baseline (data not shown). By contrast, the gene expression levels of *EP300*, as predicted with the prediction model for the small intestine, the liver and the adipose tissue, were significantly associated with the changes in body weight (F = 4.14, *p* = 0.016), BMI (F = 4.48, *p* = 0.012), total cholesterol levels (F = 6.22, *p* = 0.002), LDL cholesterol levels (F = 12.53, *p* < 0.001) and TG levels (F = 4.01, *p* = 0.02) after 6 months of AP treatment. Post-hoc analysis revealed that patients with the lowest levels of *EP300* expression showed higher increases in the abovementioned anthropometric and metabolic traits (*p* < 0.05) (Figure 4). The increase in body weight and the BMI was almost two times higher in these patients (weight increase of 14.56%; BMI increase of 15.05%) than in individuals with medium (weight increase of 7.07%, *p* = 0.006; BMI increase of 7.09%, *p* = 0.004) or high *EP300* expression (weight increase of 8.76%, *p* = 0.054; BMI increase of 8.64%, *p* = 0.029). Regarding the lipid metabolic parameters, subjects with low *EP300* expression levels showed an increase in cholesterol concentrations (22.81%) that was four times higher than those observed in patients with medium (5.08%, *p* = 0.002) or high gene expression levels (5.43%, *p* = 0.002). Furthermore, those with low *EP300* expression levels presented an increase in LDL cholesterol levels (36.81%) that was almost 12 times higher than that of patients with medium (3.16%, *p* < 0.001) or high (3.06%, *p* < 0.001) *EP300* expression. Moreover, the rise in TG concentrations was more than 2.5 times higher in individuals with the lowest *EP300* expression levels (69.98%) compared to the other participants (medium *EP300* expression: 25.75%, *p* = 0.019; high *EP300* expression: 28.27%, *p* = 0.032).

## Discussion

While accumulating evidence has linked AP therapy to metabolic disturbances, the mechanistic bases and susceptibility factors of these side effects remain unclear. In the present study, we performed an integrated analysis of gene expression profiles in four different mouse tissues after treatment with risperidone or olanzapine to identify important genes involved in the development of metabolic alterations. The key genes identified were further assessed in a naturalistic cohort of patients with FEP who were being treated with APs. Our findings provide new insights into the molecular mechanism involved in AP-induced metabolic adverse events, suggesting new candidate genes for future pharmacogenetic studies.

We first performed a WGCNA of gene expression in mice that allowed us to identify several modules of co-expressed genes that were significantly associated with risperidone or olanzapine treatment in each tissue. This approach considers the overall gene–gene correlation structure instead of focusing on individual genes, providing modules of highly interconnected genes that are enriched with genes involved in known biological pathways (Zhao et al., 2010). In our study, we found that the genes in the modules were related to biological functions that were similar for the two APs, including a high number of processes related to energy metabolism, hormone regulation and the control of vascular tone, among others. The modulation of these processes by APs at a transcriptomic level may lead to the development of AP-induced metabolic disturbances.

Most proteins carry out their functions by interacting with other components that are found either in the same cell or among different cells and even among different tissues (Barabási et al., 2011). We found that the genes of these metabolic-related pathways were highly interconnected, as demonstrated by the PPI networks constructed for each AP. The risperidone and olanzapine networks were merged to identify the overlapping genes in order to obtain a general picture of the molecular effects of both APs. The central gene of this merged network was *EP300*, which showed connections with almost all the other genes in the network. In addition, *EP300* was found to be one of the most important hub genes in the networks constructed independently for each AP. In a PPI network, nodes with a higher degree of connectivity are considered central proteins of functional importance that are essential for the maintenance of the entire network (Barabási et al., 2011). Thus, our findings suggested that *EP300* could be a crucial gene in the downstream mechanism of APs that leads to metabolic dysregulation. *EP300* encodes the protein p300, which functions as an acetyltransferase for histones and other proteins, thereby regulating transcriptional activity (Yao et al., 2018). This protein is involved in the downstream signaling of glucagon and insulin and participates in the regulation of energy homeostasis in major metabolic organs. Therefore, it has a crucial role in maintaining blood glucose levels in both fasting and postprandial states through the regulation of glycolysis, gluconeogenesis and glycogen synthesis (He et al., 2012; Wondisford et al., 2014; Huang et al., 2018; Yano et al., 2018; Yao et al., 2018; Namwanje et al., 2019). In addition, p300 is important for the maintenance of β-cell function, promotes glucose-induced insulin secretion and is associated with the disruption of insulin signaling in obesity (Mosley et al., 2004; Bompada et al., 2016; Cao et al., 2017; Wong et al., 2018). Therefore, p300 has been proposed to be a prime factor leading to the development of hyperglycemia, impaired insulin levels and insulin resistance (Cao et al., 2017; Yao et al., 2018), which frequently occur in individuals treated with second-generation APs (Newcomer et al., 2002; Henderson et al., 2005; Teff et al., 2013). Interestingly, a previous study revealed that some T2D-associated genetic variants identified by GWAS were located in sequences encoding protein binding sites, including those for p300 (Cheng et al., 2017). In addition, as observed in our study, earlier analyses have revealed *EP300* and its paralog *CBP* to be the most connected genes of the networks associated with T2D (Morris et al., 2012; Randhawa et al., 2013).

By regulating gene expression, p300 is also involved in the regulation of lipid homeostasis and adipogenesis. Thus, p300 activity may enhance triglyceride synthesis and alter lipid export, thereby increasing body weight and fat mass (Yao et al., 2018; Zhou et al., 2020). APs are associated with a higher risk of these metabolic disturbances, as patients on AP treatment frequently experience dyslipidemia, mainly due to an elevation in TG levels, as well as body weight increases and central obesity (Bioque et al., 2018; Nicol et al., 2018; Wedervang-Resell et al., 2020).

We further explored the reliability of *EP300* as a candidate gene for AP-induced metabolic dysregulation in a naturalistic cohort of patients with FEP receiving APs. To this end, we used the genetically predicted gene expression levels to assess the influence of *EP300* on different anthropometric and metabolic traits. Gene expression is an intermediate phenotype between genetic variability and disease manifestation. Most disease-associated genetic variants are located in non-coding regions, suggesting that these variants may exert their effects by modulating gene expression (Nicolae et al., 2010). In this scenario, a new promising approach has been developed that enables the prediction of gene expression based on genetic data. The imputed gene expression can then be used in place of genotypes to assess the effect of genes on a given phenotype (Gamazon et al., 2015). This strategy has been previously used to identify candidate genes associated with schizophrenia, attention deficit hyperactivity disorder and other complex traits (Gusev et al., 2018; Liao et al., 2019; Lindström et al., 2019; Wu et al., 2021). Here, we found that the predicted expression levels of *EP300* in different metabolic-related tissues of patients with FEP receiving APs were significantly associated with increases in body weight, BMI, total cholesterol levels, LDL cholesterol levels and TG concentrations at the 6-months follow-up. Although metabolic alterations have been described in schizophrenia patients even in the absence of AP treatment, we found no association between the genetically predicted *EP300* expression levels and the metabolic status of FEP patients at baseline, suggesting that this gene might be related to the AP effects instead of being a risk factor for the metabolic dysregulation associated with the disease. Thus, the genetic background would affect *EP300* expression levels which in turn could make patients more susceptible to the effects exerted by AP on this gene. Hence, these results give more support to the findings obtained in mice and strongly indicate the influence of *EP300* on AP-induced metabolic impairment.

Previous studies have found that common and rare variants of *EP300* are associated with schizophrenia risk (Ripke et al., 2014; Girard et al., 2015). However, to the best of our knowledge, this is the first time this gene has been associated with AP treatment. To date, the main mechanism of action used to explain the AP-induced metabolic adverse effects is the blockade of DRD2, H1R and 5-HT_2C_ receptors, leading to increased food intake and other metabolic impairments (De Hert et al., 2012; Reynolds and McGowan, 2017). However, a more complicated mechanism is expected to explain these effects. Recently, Chen et al. (2020) proposed a molecular mechanism of olanzapine-induced hyperphagia and obesity that could be in agreement with our results. They reported that the antagonistic effect of olanzapine on H1R activates GHSR1a (ghrelin receptor) downstream signaling in hypothalamic neurons that includes AMPK-FOXO1-pCREB signaling, which involves p300. This action increases the expression of neuropeptide Y, leading to excessive food intake and weight gain (Chen et al., 2020). Our results suggested that the regulation of *EP300* by APs in other tissues could also contribute to the appearance of metabolic disturbances. Further studies are needed to elucidate the pleiotropic effect of *EP300* on schizophrenia and AP effects and to disentangle the specific role of this gene in the mechanism of action of APs and, consequently, in AP-induced metabolic dysregulation.

The main strength of our study was the translational design, which included the integration of transcriptomic data from various mouse tissues after treatment with different APs and the subsequent exploration of the *EP300* effects in a human cohort. Nevertheless, some limitations should be considered when interpreting our findings. First, the strategy used to select the biological processes that could be related to AP-induced metabolic disturbances might have been affected by bias and, therefore, we cannot rule out the possible contribution of other genes that were not included in our analysis. Secondly, although drug dosage regimens were selected based on previous studies, here we do not control for drug plasma levels as we were unable to obtain enough blood volume of each animal to measure plasmatic drug levels with the methodology available in our facilities. Besides, the lack of a mouse group treated with an AP with a low risk of metabolic dysregulation meant that the specific effect of high and intermediate risk APs could not be explored. In addition, as no significant differences were observed in the metabolic parameters assessed between treatment groups in mice, we were not able to correlate these variables with AP-induced gene expression changes. This lack of significant differences may be due to the experimental design, in which mice were fed using a standard chow diet. Although a high-fat diet could mimic better the clinical effects of APs on metabolic parameters, which are in part due to changes in the food intake profile, we maintain the animals on a standard diet to test specifically the AP-induced gene expression changes, without the influence of external factors. Despite these limitations, we could ensure the influence of the key gene identified on metabolic impairment by assessing the effect of the genetically predicted gene expression levels in a human cohort. This exploration was conducted in a cohort of patients in a naturalistic study, and hence the sociodemographic and clinical characteristics of these individuals, such as the type of antipsychotic prescribed, could have affected the parameters assessed. For this reason, as the sample size did not allow the study of subgroups stratified by AP treatment, the statistical analysis was adjusted for several variables, including gender, age and AP potency. However, other confounders such as diagnosis, concomitant treatment or diet that we did not control for could have had an effect on the metabolic traits assessed. Finally, the prediction of the gene expression levels in this population was limited by the prediction models currently available, which in the future could include more genetic variants based on scientific advances. Hence, our results should be interpreted as exploratory and require replication.

In summary, our findings provide novel insights into the molecular mechanisms involved in the metabolic side effects of APs. Based on our results, we propose that the dysregulation of *EP300* functioning could be important in the development of AP-induced metabolic disturbances. Hence, both *EP300* and the genes involved in the *EP300* network could be considered potential candidate genes for future pharmacogenetic studies on these side effects of APs.

## Data Availability

The datasets presented in this study can be found in online repositories. The names of the repository/repositories and accession number(s) can be found below: NCBI GEO, accession no: GSE180473.
