# Supplementary material for: Identification of EP300 as a Key Gene Involved in Antipsychotic-Induced Metabolic Dysregulation Based on Integrative Bioinformatics Analysis of Multi-Tissue Gene Expression Data
Source: Front Pharmacol. 2021 Aug 13;12:729474. doi: 10.3389/fphar.2021.729474 (PMC8414590; doi:10.3389/fphar.2021.729474)
Supplement: Supplementary file 3 [file DataSheet1.PDF]

Supplementary material

MATERIALS AND METHODS

Animals and drug treatment

C57BL/6JOlaHsd male and female mice were purchased at 6 weeks of age from Harlan Laboratories (Sant Feliu de Codines, Spain) and housed in the laboratory animal center of the Faculty of Medicine at the University of Barcelona. Mice were maintained at 22ºC on a 12-hour alternating light/dark cycle with *ad libitum* access to food and water, using a standard chow diet.

After allowing the animals to acclimatize for at least ten days, they were randomly divided into three experimental groups: one received a daily subcutaneous (0.25 ml, 25G needle) dose of vehicle (saline containing 5% dimethylsulfoxide (DMSO) and 5% Tween 20), one received risperidone (1 mg/kg), and one received olanzapine (3.5 mg/kg) chronically for 28 days. Drug doses were chosen based on previous studies (Mas et al., 2016; Chen et al., 2018; May et al., 2019). Risperidone and olanzapine (Sigma Aldrich, St Louis, MO, USA) were dissolved in saline containing 5% dimethylsulfoxide (DMSO) and 5% Tween 20. Each group included 8 animals (4 males and 4 females).

All animal-related procedures were performed in accordance with the European Union guidelines for the care and use of laboratory animals and were approved by the Animal Care Committee of the University of Barcelona and by the Department of the Environment of the Generalitat de Catalunya.

Metabolic assessment in mice

Several metabolic parameters (including body weight, blood glucose levels, total cholesterol levels and triglyceride levels) were assessed in all mice at baseline (one week before the start of AP treatment) and after 28 days of treatment. Blood glucose levels were measured in the morning, after 6 hours of fasting, using a glucometer AlphaTRAK® 2 (Zoetis, Parsippany, NJ, USA), which uses coulometric technology to measure glucose in whole blood. Following the manufacturer's recommendations, the glucometer was calibrated with the manufacturer's control sample every time a new package of strips was opened. AlphaTRAK® 2 was set to "Dog Code" as indicated for its use in C57BL/6J mice (Zoetis, 2015). Blood samples were collected through tail-vain puncture by using AlphaTRAK® animal lancets (Zoetis); 0.3µl of whole blood was needed for the measurement.

Once the fasting glucose measurement was obtained, blood samples were collected to assess the levels of total cholesterol and triglycerides. For the evaluation at baseline, the distal tip (1 - 1.5 mm) of the tail was removed using a sterile scalpel blade. Blood samples (80-100ul) were collected by gently milking the tail from the base to the tip into Eppendorf tubes with heparin (40UI) and blow flow was stopped applying pressure with sterile gauze to achieve hemostasis. This procedure took less than 1 minute. For the blood collection after 28 days of treatment, the animal had already been sacrificed previously by decapitation. Then, blood was centrifugated at 4000 rpm (1252 g) during 10 minutes and the supernatant was collected. The levels of cholesterol and triglycerides were

measured using the corresponding assay kit (Cholesterol Liquid and Triglycerides Liquid; Química Clínica Aplicada S.A., Amposta, Spain). Absorbance was measured at 505 nm using a microplate reader Tecan Spark® (Tecan, Männedorf, Switzerland). Each measure was done at least in duplicate.

Tissue collection, RNA isolation and microarray hybridization

After 28 days of treatment, animals were sacrificed by decapitation 1 hour after the last injection. Then, their tissues were rapidly removed and placed on ice. In the present study, four tissues that are potentially relevant for the development of AP-induced metabolic adverse events were used: the liver, the pancreas, visceral adipose tissue (obtained from the gonadal fat pads) and the striatum (dissected out of the brain). All tissues were immediately frozen in liquid nitrogen and preserved at -80ºC until analysis. The tissue samples were homogenized in Trizol reagent (Life Technologies, Foster City, CA, USA) and total RNA was isolated following the manufacturer's instructions. The RNA samples were then further purified using the miTotal RNA Extraction Miniprep System (Viogene Biotek Corp, New Taipei City, Taiwan). RNA quantity and quality were determined using a NanoDrop ND-2000 spectrophotometer (NanoDrop, Wilmington, DE, USA). An Agilent 2100 Bioanalyzer (Agilent Technologies, Palo Alto, CA, USA) was used to assess the purity and integrity of the RNA.

A total of 1200 ng of purified RNA from each of the samples was submitted to the Kompetenzzentrum für Fluoreszente Bioanalytik Microarray Technology (KFB, BioPark Regensburg GmbH, Regensburg, Germany) for labeling and hybridization to microarray plates. Two different protocols were used for sample processing based on the RNA quality and amount. Samples from the striatum and the liver were prepared with the Affymetrix GeneChip WT PLUS Reagent Kit (Affymetrix, Inc., Santa Clara, CA, USA), whereas samples from the pancreas and adipose tissue were prepared using the Affymetrix GeneChip WT Pico Reagent Kit (Affymetrix, Inc., Santa Clara, CA, USA). The resulting cDNA was hybridized to the Affymetrix mouse Clariom S Arrays (Affymetrix, Inc., Santa Clara, CA, USA), which comprises more than 221 000 probes targeting more than 22 000 genes.

Microarray data analysis

Full details of the extraction, labeling and hybridization protocols as well as the raw array data (.cel files) and the pre-processed data matrix are available at the Gene Expression Omnibus database (http://www.ncbi.nlm.nih.gov/geo/; accession number GSE180473).

Pre-processing

The pre-processing of microarray data was performed using the Babelomics 5.0 suite (http://www.babelomics.org/) (Alonso et al., 2015). The data were standardized using robust multichip analysis. Multiple probes mapping to the same gene were merged, using the average as the sum of the hybridization values.

Weighted gene co-expression network analysis (WGCNA)

WGCNA identifies gene correlation patterns across different samples and groups highly co-expressed genes into modules that can be related to clinical traits (Langfelder and Horvath, 2008). Here, the WGCNA R package was used to identify gene co-expression

modules (https://cran.r-project.org/web/packages/WGCNA/index.html). First, a matrix of pairwise correlations between all pairs of genes across all samples was constructed. Next, a soft-thresholding power of 9 was used to obtain an adjacency matrix, a measurement of topology similarity, which was then transformed into a topological overlap matrix (TOM) and the corresponding dissimilarity. Afterwards, the dissimilarity matrix was used to construct a hierarchical clustering dendrogram whose branches were cut using the dynamic tree-cutting algorithm to obtain different modules of co-expressed genes. The above steps were performed using the automatic network construction and module detection function (*blockwiseModules* in WGCNA), with the following parameters: a minModuleSize of 30; a reassignThreshold of 0; and a mergeCutHeight of 0.25. The association of the different modules with AP treatment (risperidone or olanzapine) was evaluated by calculating the correlation between the module eigengenes (MEs, the first principal component of each module) and the treatment status (risperidone vs vehicle or olanzapine vs vehicle). Modules with a significant correlation (p-value < 0.05) were selected for further analysis. For all the genes included in each significant module, the gene significance (GS, representing the association between each gene expression and AP treatment) and its module membership (MM, the correlation between gene expression and the module eigengene) were calculated.

Gene set enrichment analysis

To provide a functional interpretation of the different transcriptional activities identified, a gene set enrichment analysis was performed using the genes included in each gene co-expression module that was significantly associated with AP treatment (risperidone or olanzapine). First, mouse genes were converted into human orthologs in order to use the most well-described human annotation databases. The analysis was conducted using FatiGO (Al-Shahrour et al., 2007), a web-based tool implemented in the Babelomics 5.0 suite that uses Fisher's exact test to identify the biological processes significantly overrepresented in a set of genes. Gene Ontology (GO) terms (Ashburner et al., 2000) were used to define the biological processes and a functional enrichment of the submitted gene lists was performed against the rest of the genome. Only functional terms with a false discovery rate (FDR)-corrected p-value < 0.05 were considered significantly enriched. The GO terms related to metabolic processes and metabolic dysregulation were then selected for further analysis. This prioritization was achieved by a final consensus among four independent researchers.

Protein-protein interaction (PPI) network construction and evaluation

Genes included in the selected GO terms were used to create a single PPI network for each AP (risperidone or olanzapine). The minimum connected network (MCN), defined as the shortest network connecting all the interacting nodes within a gene list, was obtained using the SNOW program (Minguez et al., 2009) implemented in the Babelomics 5.0 suite. Briefly, we used the curated interactome (validated by at least two independent methods) and allowed the inclusion of extra nodes that were not included in our list and that connected two or more nodes in the list. Network enrichment analysis was performed to test whether the parameters that described the network were beyond random expectations or not. The parameters analyzed were the degree of connectivity, the clustering coefficient and the betweenness centrality. The PPI networks obtained were visualized and analyzed using Cytoscape 3.7.2 (Shannon et al., 2003). A single PPI network integrating both APs was also constructed by merging the networks obtained independently for risperidone and olanzapine using the intersection option in Cytoscape.

For each node, the degree of connectivity, which accounts for the number of edges or direct interactions a particular node has, was calculated to identify the candidate hub genes from the constructed PPI networks.

Exploration of the hub genes in a naturalistic cohort of patients with FEP

Participants

From the original sample of 335 patients with FEP recruited in the project "Phenotype–genotype and environmental interaction: application of a predictive model in first-episode psychosis, FIS PI080208" (known as the PEPs study from the Spanish abbreviation for first-episode psychosis), 302 participants were prescribed at least one second-generation AP during the follow-up period. Of these, 226 (age 23.6 ± 6.0 years; 66.8% were males) provided biological samples for genotyping and completed the six-month follow-up period. As this was a naturalistic study, there were no specific guidelines for treatments (drugs and/or psychotherapy). During the follow-up period, about 40% of the patients were treated with olanzapine or clozapine, which have the highest risk of inducing weight gain or worsening metabolic parameters, while more than 50% were administered APs with an intermediate or low risk of causing such side effects, with risperidone and aripiprazole being the most frequently prescribed in this group.

A complete description of the protocol for the PEPs study has been published (Bernardo et al., 2013; Bioque et al., 2016). The study was approved by the ethics committees of all the participating clinical centers. Informed consent was obtained from all the participants. In the case of children under 16 years of age, parents or legal guardians gave written informed consent before study participation, while the patients themselves also agreed to participate.

Metabolic assessment

Several anthropometric and metabolic traits were assessed at each visit during the PEPs study (Bioque et al., 2018; Gassó et al., 2020). The metabolic-related variables measured were body weight, body mass index (BMI), blood glucose levels, total cholesterol levels, low-density lipoprotein (LDL) cholesterol levels, high-density lipoprotein (HDL) cholesterol levels and triglyceride (TG) levels. For the present study, we calculated the percentage of change for each parameter between the six-month follow-up and the baseline visit.

Sample collection, genotyping and SNP imputation

Blood samples from all participants were collected in EDTA tubes (K2EDTA BD Vacutainer; Becton Dickinson, Franklin Lakes, NJ, USA). Genomic DNA was extracted using the MagNA Pure LC DNA isolation Kit III and an LC MagNA Pure system (Roche Diagnostics GmbH, Mannheim, Germany). The concentration and quality of DNA were measured using a NanoDrop 2000 (NanoDrop, Wilmington, DE, USA). Samples from all the individuals in the study were genotyped at the Centro Nacional de Genotipado (CeGen, Santiago de Compostela, Spain), using the Affymetrix Axiom Spain Biobank Array containing probes for 758 740 SNPs. The genotyping data were then called using the Axiom Analysis Suite (Mas et al., 2020). The genotyping data were submitted to the Michigan Imputation Server (Das et al., 2016), following the standard pipeline for the

Minimac4 software and setting the European population reference from the GRCh37/hg19 build and Eagle v2.4 phasing.

Gene expression prediction

Genotyping data were used to predict the genetically regulated gene expression levels for each individual through the gene expression imputation method, PrediXcan (Gamazon et al., 2015). PrediXcan uses reference transcriptome datasets from the Genotype-Tissue Expression (GTEx) Project to train additive models of gene expression levels. After that, these models are used to estimate the genetically regulated expression of genes that constitutes the prediction of expression levels using multiple SNPs. These predictive models are used to 'impute' gene expression in local data. Gene expression of the previously identified hub genes was predicted using the multivariate shrinkage (mashr) models built from the GTEx version 8 available at https://predictdb.org/. We predicted expression levels in five different tissues that are potentially relevant for the development of AP-induced metabolic adverse events, including the small intestine, the pancreas, the liver, visceral adipose tissue and subcutaneous adipose tissue, following the standard procedure found at https://github.com/hakyimlab/PrediXcan.

Statistics

Data were analyzed using IBM SPSS Statistics version 20.0 (IBM Corp, Chicago, IL, USA). Normality was assessed using Kolmogorov-Smirnov test. The predicted gene expression levels of the *EP300* gene were categorized as low, medium or high according to the three values of gene expression obtained in each tissue (-0.13, -0.07 and 0.00, respectively). Differences in metabolic variables between these categories were tested using analysis of variance. The analysis was adjusted for sociodemographic and clinical variables that might affect the parameters assessed, including gender, age, and the type of AP according to its potency (high, low, or no risk of increasing weight or worsening metabolic parameters). A further pairwise comparison analysis was performed using Bonferroni's post-hoc test.

SUPPLEMENTARY FIGURES

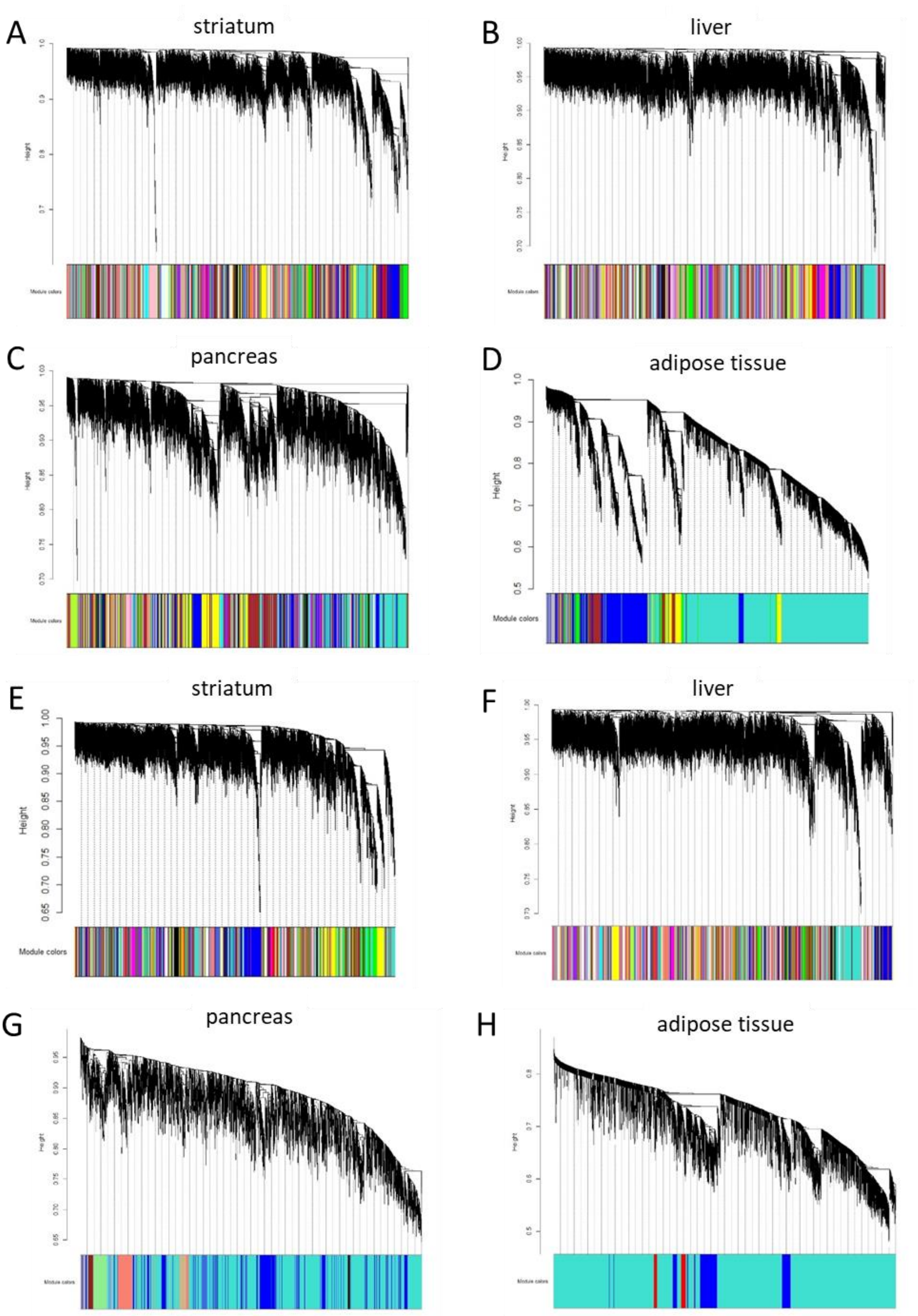

Supplementary Figure 1. Clustering dendrogram showing the co-expression modules identified in the striatum, liver, pancreas and adipose tissue of risperidone-treated mice (A-D) and olanzapine-treated mice (E-H). Each module is represented by a different color in the corresponding color bar below the dendrogram.

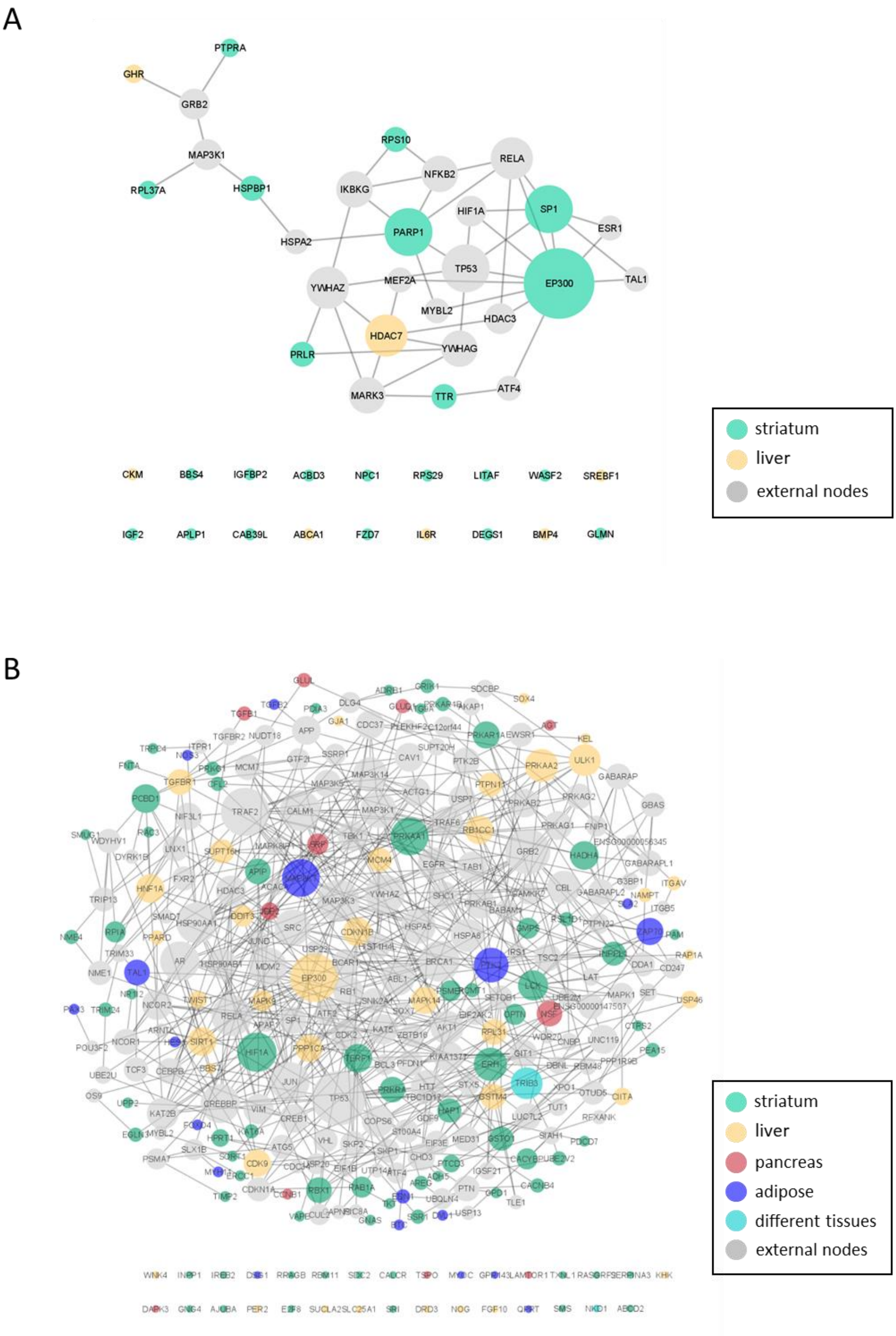

Supplementary Figure 2. Protein-protein interaction networks constructed using the genes included in the selected GO terms that were significantly enriched in the co-expression modules identified for risperidone (A) and olanzapine (B). Node size represents the number of connections (degree) of each gene.

REFERENCES

Al-Shahrour, F., Minguez, P., Tárraga, J., Medina, I., Alloza, E., Montaner, D., et al. (2007). FatiGO +: A functional profiling tool for genomic data. Integration of functional annotation, regulatory motifs and interaction data with microarray experiments. *Nucleic Acids Res.* 35, 91–96. doi:10.1093/nar/gkm260.

Alonso, R., Salavert, F., Garcia-Garcia, F., Carbonell-Caballero, J., Bleda, M., Garcia-Alonso, L., et al. (2015). Babelomics 5.0: Functional interpretation for new generations of genomic data. *Nucleic Acids Res.* 43, W117–W121. doi:10.1093/nar/gkv384.

Ashburner, M., Ball, C., Blake, J. A., Botstein, D., Butler, H., Cherry, J. M., et al. (2000). Gene ontology: Tool for the identification of biology. *Nat. Genet.* 25, 25–29. doi:10.1038/75556.

Bernardo, M., Bioque, M., Parellada, M., Ruiz, J. S., Cuesta, M. J., Llerena, A., et al. (2013). Assessing clinical and functional outcomes in a gene–environment interaction study in first episode of psychosis (PEPs). *Rev. Psiquiatr. y Salud Ment. (English Ed.* 6, 4–16. doi:10.1016/j.rpsmen.2012.11.001.

Bioque, M., García-Portilla, M. P., García-Rizo, C., Cabrera, B., Lobo, A., González-Pinto, A., et al. (2018). Evolution of metabolic risk factors over a two-year period in a cohort of first episodes of psychosis. *Schizophr. Res.* 193, 188–196. doi:10.1016/j.schres.2017.06.032.

Bioque, M., Llerena, A., Cabrera, B., Mezquida, G., Lobo, A., González-Pinto, A., et al. (2016). A pharmacovigilance study in first episode of psychosis: psychopharmacological interventions and safety profiles in the PEPs Project. *Int. J. Neuropsychopharmacol.* 19, 1–10. doi:10.1093/ijnp/pyv121.

Chen, C. H., Shyue, S. K., Hsu, C. P., and Lee, T. S. (2018). Atypical antipsychotic drug olanzapine deregulates hepatic lipid metabolism and aortic inflammation and aggravates atherosclerosis. *Cell. Physiol. Biochem.* 50, 1216–1229. doi:10.1159/000494573.

Das, S., Forer, L., Schönherr, S., Sidore, C., Locke, A. E., Kwong, A., et al. (2016). Next-generation genotype imputation service and methods. *Nat. Genet.* 48, 1284–1287. doi:10.1038/ng.3656.

Gamazon, E. R., Wheeler, H. E., Shah, K. P., Mozaffari, S. V., Aquino-Michaels, K., Carroll, R. J., et al. (2015). A gene-based association method for mapping traits using reference transcriptome data. *Nat. Genet.* 47, 1091–1098. doi:10.1038/ng.3367.

Gassó, P., Arnaiz, J. A., Mas, S., Lafuente, A., Bioque, M., Cuesta, M. J., et al. (2020). Association study of candidate genes with obesity and metabolic traits in antipsychotic-treated patients with first-episode psychosis over a 2-year period. *J. Psychopharmacol.* 34, 514–523. doi:10.1177/0269881120903462.

Langfelder, P., and Horvath, S. (2008). WGCNA: An R package for weighted correlation network analysis. *BMC Bioinformatics* 9, 559. doi:10.1186/1471-2105-9-559.

Mas, S., Boloc, D., Rodríguez, N., Mezquida, G., Amoretti, S., Cuesta, M. J., et al. (2020). Examining gene-environment interactions using aggregate scores in a first-episode psychosis cohort. *Schizophr. Bull.* 46, 1019–1025.

doi:10.1093/schbul/sbaa012.

Mas, S., Gassó, P., Boloc, D., Rodriguez, N., Mármol, F., Sánchez, J., et al. (2016). Network analysis of gene expression in mice provides new evidence of involvement of the mTOR pathway in antipsychotic-induced extrapyramidal symptoms. *Pharmacogenomics J.* 16, 293–300. doi:10.1038/tpj.2015.48.

May, M., Beauchemin, M., Vary, C., Barlow, D., and Houseknecht, K. L. (2019). The antipsychotic medication, risperidone, causes global immunosuppression in healthy mice. *PLoS One* 14, e0218937. doi:10.1371/journal.pone.0218937.

Minguez, P., Götz, S., Montaner, D., Al-Shahrour, F., and Dopazo, J. (2009). SNOW, a web-based tool for the statistical analysis of protein-protein interaction networks. *Nucleic Acids Res.* 37, W109–W114. doi:10.1093/nar/gkp402.

Shannon, P., Markiel, A., Ozier, O., Baliga, N. S., Wang, J. T., Ramage, D., et al. (2003). Cytoscape: a software environment for integrated models. *Genome Res.* 13, 2498–2504. doi:10.1101/gr.1239303.

Zoetis 2015. AlphaTRAK2 blood glucose monitoring system user guide and package insert. Parsippany (NJ): Zoetis Services
